# Study on the Role of MicroRNA-214 in the Rehabilitation of Cartilage in Mice with Exercise-Induced Traumatic Osteoarthritis

**DOI:** 10.3390/cimb44090281

**Published:** 2022-09-07

**Authors:** Hong Cao, Xuchang Zhou, Hui Li, Miao Wang, Wei Wu, Jun Zou

**Affiliations:** 1 School of Kinesiology, Shanghai University of Sport, Shanghai 200438, China; 2 School of Sport Medicine and Rehabilitation, Beijing Sport University, Beijing 100084, China

**Keywords:** post-traumatic osteoarthritis, treadmill exercise, articular cartilage, miR-214

## Abstract

This study aimed to explore the possible relationship between the expression of Micro RNA-214 (miR-214) and the pathogenesis and recovery in mice with post-traumatic osteoarthritis (PTOA). In this study, 40 male C57BL/6 mice were randomly divided into five groups: model control (MC) group, model (M) group, rehabilitation control (RC) group, model + rehabilitation (M + R) group, and model + convalescent (M + C) group. Four weeks of high-intensity treadmill exercise (HITE) and 4 weeks of moderate-intensity treadmill exercise (MITE) were implemented for PTOA modeling and rehabilitation, respectively. In vitro, 10% elongation mechanical strain was used for IL-1β stimulated chondrocytes. We found that compared with the MC group, there was a significant increase in the aspect of inflammation and catabolism while a dramatic fall in miR-214 expression was observed in the M group. After the 4 weeks of MITE, the level of inflammation and metabolism, as well as miR-214 expression, was partially reversed in the M + R group compared with the M + C group. The expression of miR-214 decreased dramatically after chondrocyte stimulation by IL-1β and then increased significantly after 10% strain was applied to IL-1β-treated cells. These results suggest that a suitable mechanical load can increase the expression of miR-214, and that miR-214 may play a chondroprotective effect in the development of OA.

## 1. Introduction

Osteoarthritis (OA) is a chronic degenerative and inflammatory joint disease characterized by the damage and degeneration of articular cartilage, abnormal subchondral bone remodeling, osteophyte formation, synovial inflammation, and angiogenesis, which usually leads to joint pain, swelling, stiffness, and limited joint movement in patients with OA [1]. As the global population ages, the number of people suffering from OA has gradually increased. It is estimated that more than 250 million people are currently affected by OA, placing a severe burden on society [2]. PTOA is a common subtype of OA which usually occurs after acute joint injury or mechanical stress overload [3]. Under normal physiological conditions, the lower limb articular cartilage is often subjected to pressure, shearing force, and other complex mechanical stimuli, maintaining the cartilage matrix in a state of a dynamic balance of catabolism and anabolism. Abnormal mechanical conditions will lead to cartilage damage: mechanical unloading will cause cartilage atrophy and thinning, and excessive mechanical load will cause cartilage damage [4,5,6]. Researchers have revealed that the knee joint cartilage bears about 10 times the mechanical load of the weight of the body during running and about 20 times during jumping [7,8,9]. Therefore, factors such as obesity, joint deformities, joint instability, or long-term, high-intensity running and jumping exercises impose excessive mechanical loads on the articular cartilage, resulting in an imbalance in cartilage matrix homeostasis (activation of catabolism and inhibition of anabolism), which eventually leads to PTOA. Our previous studies have demonstrated that high-intensity treadmill running can cause cartilage damage in mice [10].

The treatment of OA, including surgery and non-surgical treatment, can only relieve the clinical symptoms or delay the pathological processes of the disease; to date, no clinical treatment can completely reverse the pathology and disease progression [11]. Surgical treatment is usually used in the late stage of OA, which is expensive and may risk complications such as postoperative infection and thrombosis [12]. Non-surgical treatments, mainly non-steroidal anti-inflammatory drugs (NSAIDs)and exercise therapy, tend to be used in the early stages of OA. In addition, NSAIDs may cause side effects such as cardiovascular risk or impaired gastrointestinal function [13,14,15]. Therefore, in recent years, studies on exercise treatment for OA have received more and more attention. As for conservative nonsurgical therapy, exercise therapy has the advantages of high acceptability and low cost. The Osteoarthritis Research Society International (OARSI) guidelines recommended exercise therapy as an effective first-line intervention for the self-management of knee OA [16,17]. Accumulated clinical studies have confirmed that exercise therapy can effectively relieve joint pain and increase the range of joint motion by enhancing the strength of muscles around the joint and correcting abnormal mechanical properties of articular cartilage [18,19,20,21]. Animal studies have also verified the rehabilitation effect of exercise therapy on OA. Moderate-intensity treadmill exercise (MITE) can increase endogenous opioid signals and reduce joint pain in mice with late-stage OA. In addition, treadmill exercise can effectively reduce subchondral bone resorption, reducing the loss of bone mass and ultimately improving the load-bearing capacity of articular cartilage in OA mice [10,22,23]. In vitro studies have also found that moderate mechanical stress can effectively reduce the expression of catabolic factor matrix metalloproteinase 13 (MMP-13) and increase the expression of anabolic factor COL-II in chondrocytes, partially reversing the metabolic phenotype of OA chondrocytes [24].

MicroRNAs (miRNAs) are a type of non-coding RNA with a length of about 18~24 nucleotides. MiRNAs can bind to the 3′-untranslated region (UTR) sequence of the target mRNA to degrade it and inhibit its translation, which hinders the expression of the target gene. MiRNAs are involved in various life processes, including apoptosis, proliferation, and differentiation [25]. Micro RNA-214 (miR-214) is located in the long non-coding Dmn3os transcription sequence; its precursor is spliced to generate two mature miRNAs, i.e., miR-214-3p and miR-214-5p [25]. MiR-214 is highly conserved among multiple species and coordinates the physiological processes involved in various diseases associated with muscles, bones, the cardiovascular system, and the nervous system [26,27,28,29]. MiR-214 is also significant in the pathological changes associated with diseases such as cancer and osteoporosis [30,31]. MiR-214 has high specificity for cartilage tissue [32]. Studies have confirmed that the abnormal expression of miR-214 can cause bone and cartilage development disorders and lead to the occurrence of OA [26,27]. Our previous studies have found that exercise therapy can act on miR-214 to prevent the occurrence and development of osteoporosis [28,29]. However, the effect of miR-214 on the development of PTOA caused by abnormal mechanical stress is still unclear, and further research is still needed. It is well known that excessive exercise may cause damage and degeneration of articular cartilage, while proper exercise can protect cartilage, thus preventing or delaying the progression of OA. In this study, we established the exercise-induced PTOA model through high-intensity treadmill exercise (HITE) and then used MITE to perform rehabilitation training on the exercise-induced PTOA model. Detection of miR-214 expression in the HITE and MITE processes, respectively, was performed. Our purpose was to explore the possible regulatory mechanism of miR-214 in an exercise-induced PTOA model.

## 2. Materials and Methods

### 2.1. Animal Models

This study was performed with the consent of the Ethical Committee of Shanghai University of Sport on the Care and Use of Animal Subjects in Research (Approval Number: 2018013). Forty C57BL/6 male mice aged 19 weeks were purchased from Shanghai Southern Model Biotechnology Co., Ltd (Shanghai, China). All mice were kept in individually ventilated cages (IVC) in the pathogen-free animal laboratory of the Shanghai University of Sport. A light control system was used to simulate the 12-h alternation of day and night. The temperature was 22 ± 2 °C and the humidity was 40% to 70% in the animal room. All the mice were weighed at weekly intervals.

### 2.2. Experimental Grouping

After 1 week of adaptive feeding, 20-week-old mice were randomly separated into five groups: model control group (MC group, n = 8), model group (M group, n = 8), rehabilitation control group (RC group, n = 8), model + rehabilitation group (M + R group, n = 8) and model + convalescent group (M + C group, n = 8). The MC group and RC group were not subjected to any treadmill intervention. The mice in the M, M + R, and M + C groups were pre-adapted to treadmill exercise at a low speed for 1 week and then subjected to HITE for 4 weeks for PTOA modeling. Later, mice in the MC and M groups were sacrificed at 25 weeks of age to verify whether the HITE could successfully establish an exercise-induced PTOA model. Subsequently, after one week of rest, when the mice were 26 weeks old, the mice in the M + R group received 4 weeks of MITE, while those in the M + C group did not receive any intervention. All mice in the RC, M + R, and M + C groups were sacrificed at 30 weeks of age to verify the rehabilitation effect of MITE on the exercise-induced PTOA mice (Table 1).

### 2.3. Treadmill Exercise Protocol

By consulting related literature and based on our previous research [30,31], the following treadmill experiment protocol was formulated (As shown in Table 2).

Pre-adaptation treadmill exercise training was implemented, lasting 1 week, five times a week, 60 min each time. The slope of the treadmill was 5°. The speed on the first day was 0–12 m/min, the second day: 0–14 m/min, the third day: 0–16 m/min, the fourth day: 0–18 m/min, and on the fifth day: 0–20 m/min.

HITE was implemented when the mice were 21 weeks old, lasting 4 weeks, 5 times a week, 80 min for each time. The slope of the treadmill was again 5°. The speed of the treadmill exercise was gradually accelerated to 20 m/min in the first 10 min, then continued 20 m/min for 60 min, and gradually decelerated to 0 m/min in the last 10 min.

MITE was implemented when the mice were 26 weeks old, lasting 4 weeks, 5 times a week, 40 min each time, and the constant treadmill speed was 8 m/min.

### 2.4. Sampling

When the HITE was completed, the mice in the MC and M groups were sacrificed. After completing the MITE, the mice in the RC, M + R, and M + C groups were sacrificed. All mice were prohibited from feeding 12 h before sacrifice. All mice were anesthetized by inhalation of isoflurane and sacrificed. The lower limbs of the mice were separated using ophthalmic surgical scissors, and all soft tissues around the knee joint were stripped, leaving the knee joint intact for micro-CT and RT-qPCR detection.

### 2.5. Micro-CT Scan

Micro-CT was used to determine the microstructure of subchondral bone. The placement angle of the knee joint had to be consistent and readjusted in the scan window to ensure the accuracy of scanning and reconstruction. When the scanning process was finished, 100 layers were selected of reconstructed femoral condyle subchondral bone, upward from the position where the femoral condyle appeared. The scanning parameters of Micro-CT were as follows: FOV/Diameter: 31,948 μm, Voxel size: 10.4 μm, Energy: 55 kv, Intensity: 72 μA, Sample time: 250,000 μs. The bone morphological indicators tested were as follows: (1) trabecular spacing (Tb.Sp), (2) trabecular thickness (Tb.Th), (3) trabecular number (Tb.N), (4) bone volume fraction (BT/TV), and (5) bone mineral density (BMD).

### 2.6. Histological Evaluation

The bone was placed in paraformaldehyde for 48 h and then transferred to the EDTA solution for decalcification. The bone was decalcified at room temperature on a shaker for about 1 month, and the decalcification solution was changed every 2 days until the bone tissue could be easily cut by a blade. When the bone was fully decalcified, the joints were soaked in 50% alcohol at room temperature for 12 h. The next day, the knee joints were dehydrated using ethanol with gradient concentrations. Then, the knee joints were subjected to xylene transparentizing and wax embedding. The popliteal side of the joint was uniformly placed in the embedding box. The knee joints were coronal sectioned at a slice thickness of 5 μm. The excess part of the wax block was cut up to the weight-bearing area of cartilage, and then the knee joint was carefully sliced coronally and stored for staining. HE and Safranin-O-Fast Green staining were used after the sections had been deparaffinized and dehydrated. The staining slices were evaluated by two raters under a 200× light microscope in strict accordance with the standards of Mankin score and OARSI score [32,33]. The average of the two scores was regarded as the final score.

### 2.7. RT-qPCR Detection

The soft tissue around the cartilage was carefully removed. The cleaned cartilage was put in a mortar and ground into powder in a mortar filled with liquid nitrogen. The total RNA was extracted according to the conventional procedure. The expressions of genes such as miR-214, β-catenin, WNT1, TNF-α, MMP13, COL2A, and ACAN was tested. U6 and β-actin were selected as miRNA and mRNA internal reference genes, respectively. All primers were designed using Premier 5.0 software. The primer sequence is shown in Table 3.

### 2.8. Cell Isolation and Culture

Three-day-old C57BL/6 mice were sacrificed for the collection of articular cartilage from knees under sterile conditions. Samples were then washed three times with cold 1× PBS solution and additional unrelated soft tissue was peeled off. The cartilage was digested in digestive DMEM solution (1% Collagenase type II and 2% Dispase II neutral protease) for 30 min and DMEM (0.5% Collagenase type II and 1% Dispase II neutral protease) for 6 h at 37 °C. After 6 h digestion, 2 mL of DMEM medium containing 10% fetal bovine serum and 1% penicillin/streptomycin was added to stop digestion. The released chondrocytes were seeded in 10 cm^2^ cell culture dishes. Cells were passaged when they reached 80–90% confluency. The third-generation cells were used for the experiment. The P3 cells were evaluated with toluidine blue O staining to ascertain that they were chondrocytes.

### 2.9. Cell Treatments Protocol

In the IL-1β stimulating groups, chondrocytes were cultured in six-well Flexcell Bio-Flex plates and stimulated with IL-1β (10 ng/mL) to simulate an OA-like inflammatory response. For the mechanical strain groups, a Flexcell-5000C Tension System was used to provide mechanical strain. The mechanical strain protocol was 10% elongation at a frequency of 0.5 Hz for 0 h, 1 h, 2 h, and 4 h respectively. The control group was cultured in six-well Flexcell Bio-Flex plates without any IL-1β stimulation or mechanical strain. Table 4 lists the detailed mechanical straining protocol parameters.

### 2.10. Cell Viability Assay

A CCK8 assay was used to determine cell viability after IL-1β treatment. A microplate reader was used for OD value detection. The detected chondrocytes were incubated in 96-well plates at a density of 1 × 10^4^/well. To ensure the accuracy of the experiment, five wells were set up. Then, IL-1β at different concentrations (0, 0.1 ng/mL, 1 ng/mL, 10 ng/mL, 20 ng/mL) was added to each well. After 72 h of IL-1β stimulation, cells were incubated with CCK8 solution (DMEM medium with 10% CCK8). The detection wavelength was 450 nm, and the reference wavelength was 570 nm. Dual-wavelength detection was used.

Cell viability = [(As − Ab)/(Ac − Ab)] × 100%, where As is the OD value of experiment wells, Ac is the OD value of control wells, and Ab is the OD value of blank control wells.

### 2.11. RT-qPCR Detection

The total RNA was extracted according to the conventional procedure. OA-related genes such as miR-214, IL-6, MMP-13, COL-II, and ACAN were detected. U6 and GAPDH were selected as internal reference genes for miRNA and mRNA. The primer sequence is shown in Table 3.

### 2.12. Western Blot Analysis

Once the mechanical strain intervention was finished, cells were washed three times with PBS solution. Next, 100 μL RIPA Lysis Buffer (Biyuntian, China) was added and the cells were placed on ice for 30 min. After centrifugation for 10 min at 14,000 rpm at 4 °C, the total protein was collected. The concentration level of protein was quantified using a BCA kit. The protein was boiled in water for 10 min and then stored at –20 degrees for later use.

Proteins were separated by SDS-PAGE (10% gels) and then transferred onto the PVDF membrane. The transferred membranes were pre-incubated in 5% skim milk powder for 1 h. Then, the blotting membranes were incubated with COL2 (Abclonal, Wuhan, China A1560), MMP13 (Thermo Fisher, Shanghai, China, MA5-14247), and β-tubulin (Abclonal, Wuhan, China, A12289) overnight at 4 °C. After washing with TBST three times for 10 min, the membranes were incubated with the secondary antibody (1:2000) for 1 h. The blots were quantified using a Tanon 5200 Multi-system (Tanon Science and Technology Co., Ltd., Shanghai, China). Image J software (National Institutes of Health, Bethesda, MD, USA) was used to analyze the protein band.

### 2.13. Data Analysis

All experimental results are shown as mean ± standard deviation (X ± SD). SPSS 20.0 software was used for subsequent data analysis. The independent sample *t*-test was used to make pairwise comparisons between the MC and M groups. The one-way analysis of variance was applied to compare the main effects of the RC, M + R, and M + C groups, and then the LSD method was subjected to post-hoc testing. *p* < 0.05 indicates that the results are statistically significant.

## 3. Results

### 3.1. The Effect of Exercise on the Weight of Mice

**Figure 1:**
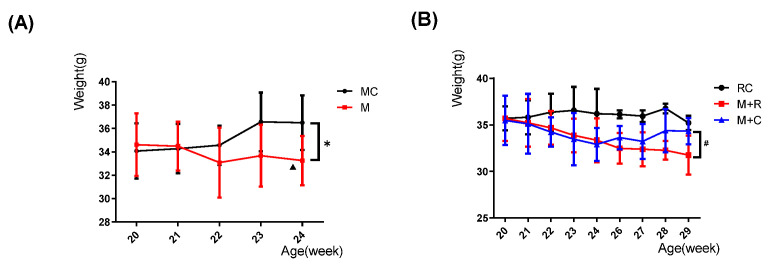
Changes in the weights of mice at different ages in each group. (**A**) Changes in the weight of mice in the MC and M groups; (**B**) Changes in the weight of mice in the RC, the M + R, and M + C groups. (n = 8, Comparison within M group: ^▲^ *p* < 0.05; comparison between MC and M groups: * *p* < 0.05; comparison between M + R and M + C groups: ^#^ *p* < 0.05).

Body weight showed an overall increasing trend in the MC group as the age increased, but without any statistical significance, while the weight of mice in the M group showed an overall downward trend, and the weight of mice at 22 and 24 weeks of age showed a decrease in prominent differences, in contrast to that of mice at 20 weeks of age (*p* < 0.05). In addition, at 24 weeks of age, the weight of the M group had a prominently lower trend compared to the MC group (*p* < 0.05) (Figure 1A).

The weight of the RC group showed an increasing trend with increasing age, although there was no statistical difference. During the HITE period, the weight in the M + R and the M + C groups showed a continuous downward trend. It is worth noting that at 24 weeks of age, the weight of the M + C group was lower than that of the RC group (*p* < 0.05), which was similar to the results in the MC and M groups. During the MITE, the weight of the M + R group started to decrease, but without statistical significance. However, after stopping the HITE, the weight of M + C group increased slightly and the weight at 29 weeks was significantly larger than in the M + R group (*p* < 0.05) (Figure 1B).

### 3.2. The Effect of Exercise on Articular Cartilage and Chondrocyte

**Figure 2:**
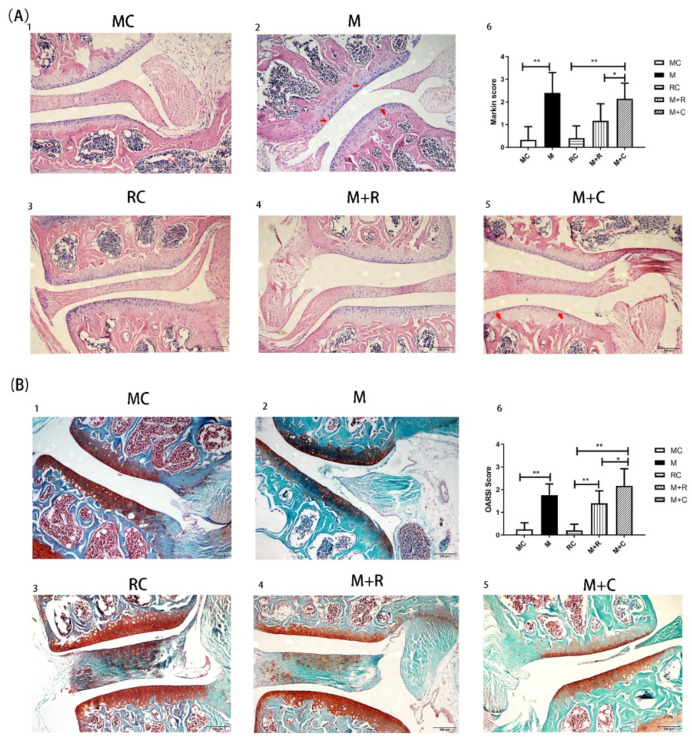
Histological evaluation of joint tissue sections by HE and safranin O staining. (**A**) HE stained histological images of the knee joints; **A(1)**: HE staining for MC group; **A(2)**: HE staining for M group; **A(3)**: HE staining for RC group; **A(4)**: HE staining for M + R group; **A(5)**: HE staining for M + C group; **A(6)**: The Mankin score of each group; (**B**) Safranin-O-Fast Green stained histological images of the knee joints of mice; **B(1)**: Safranin-O-Fast Green staining for MC group; **B(2)**: Safranin-O-Fast Green staining for M group; **B(3)**: Safranin-O-Fast Green staining for RC group; **B(4)**: Safranin-O-Fast Green staining for M + R group; **B(5)**: Safranin-O-Fast Green staining for M + C group; **B(6)**: The Mankin score of each group. The red arrow indicates the site of cartilage damage. (n = 8, * *p* < 0.05, ** *p* < 0.01.).

Sections observed with an intact joint structure under a light microscope were selected for HE and Safranin-O-Fast Green staining. Hematoxylin dye can combine with the nucleus to appear blue, while eosin dye can combine with cytoplasm and the extracellular matrix to appear red. Safranin O dye is weakly alkaline and appears red when combined with basophilic cartilage tissue, while the fast green dye is acidic and appears blue or green when combined with eosinophilic bone tissue. As shown in Figure 2(A1), the surface of the articular cartilage of mice in the MC group (with no intervention) was smooth and evenly stained, and the chondrocytes were neatly arranged. As shown in Figure 2(B1), the knee cartilage in the MC group was evenly stained with intact cartilage volume and without obvious defects or calcification in the cartilage. After the HITE period in the M group (Figure 2(A2)), there were irregular fissures on the cartilage surface. The chondrocytes were arranged in a disorderly manner and the cells were dramatically reduced in contrast to the MC group. The volume of the safranin O-stained cartilage of the M group was less than that in the MC group, indicating that there was a large cartilage volume loss in the former group. Blue staining appeared in the red cartilage area, indicating that there was endochondral ossification in the M group (Figure 2(B2)).

After 4 weeks of HITE + 4 weeks of convalescence, the surface of the articular cartilage in the M + C group was rough and presented small cracks (Figure 2(A5)). The chondrocytes were arranged unevenly and their numbers were significantly reduced. Additionally, the red staining area in the cartilage of the M + C group was small, and there was still bone tissue stained with green inside the cartilage (Figure 2(B5)). This revealed that there was still significant cartilage volume loss and calcification in the M + C group. After 4 weeks of HITE + 4 weeks of MITE, there were only a few cracks on the cartilage surface of the M + R group (Figure 2(A4)), and the chondrocytes were neatly arranged, suggesting that cartilage damage was less severe compared to the M + C group. Figure 2(B4) also shows that the volume of red tissue in the cartilage was significantly greater than in the M + C group. There was less blue bone tissue in the cartilage. This indicated that although there was partial cartilage volume loss and mild intrachondral calcification in the M + R group, the severity of cartilage damage was lower than that in the M + C group.

Subsequently, two independent observers evaluated the knee joint slices under a 200× light microscope, following the Mankin and OARSI scoring standards (Figure 2 (A6,B6)). The results from both scoring standards showed that the scoring value of cartilage in the M group was significantly higher than in the MC group (*p* < 0.05). The histological staining results and scores proved that the HITE led to cartilage injury, which successfully established the PTOA model. Furthermore, the Mankin and OARSI scores in the M + R group were significantly lower than in the M + C group (*p* < 0.05), while the Mankin score in the M + C group was substantially higher than in the RC group (*p* < 0.01). This proved that MITE could alleviate cartilage surface damage and chondrocyte arrangement disorder. It also partially alleviated cartilage loss and calcification compared to that observed after 4 weeks of rest.

### 3.3. The Effect of Exercise on the Subchondral Bone of Femoral Condyle

**Figure 3:**
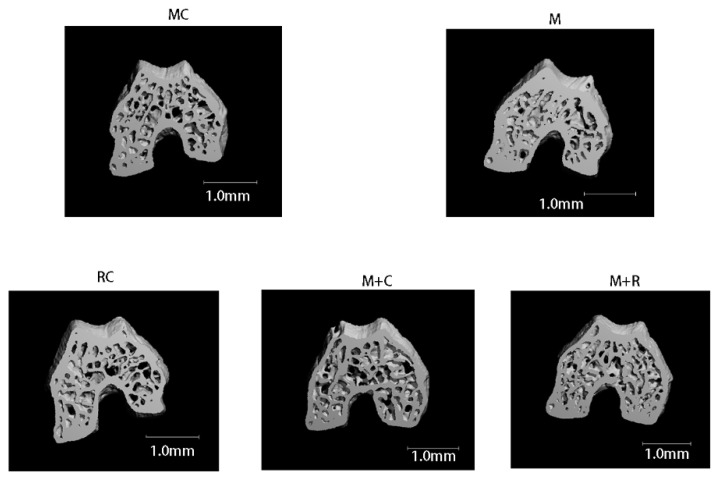
3D reconstruction of the subchondral bone of the femoral condyle by micro-CT.

Images of the 100 upper layers of the femoral condyle were captured to reconstruct the subchondral bone of the knee joint to obtain bone morphological indicators. The three-dimensional reconstruction of the subchondral bone microstructure is shown in Figure 3. The bone trabeculae in the MC and M + R groups were regular in shape and neatly arranged. Compared with the MC group, the trabecular bone cortex of the M group was thickened with significantly increased bone mass. Moreover, in contrast to the M + R group, the number of trabecular bones in the M + C group was significantly less, and the arrangement was uneven.

As shown in Table 5, after 4 weeks of HITE, the BV/TV of the femoral condyle subchondral bone of the M group was significantly higher than that of the MC group (*p* < 0.05), while the number of Trabecular (Tb.N), bone mass and bone mineral density (Tb.Th) and BMD values had a higher trend than those in the MC group, albeit without significant differences. Furthermore, after 4 weeks of MITE, the values of bone mineral density (BMD), Tb.N, BV/TV of the M + R group were greatly improved and higher than those of the M + C group, while the average width of the medullary cavity between trabeculae (Tb.Sp) was significantly lower than that of the M + C group (*p* < 0.05).

When osteoporosis occurs, the Tb.N, Tb.Th and BMD values decrease. In our study, the value of Tb.Sp increased, suggesting the increased bone resorption.

### 3.4. The Effect of Exercise on Articular Cartilage Metabolism and Inflammation Expression

**Figure 4:**
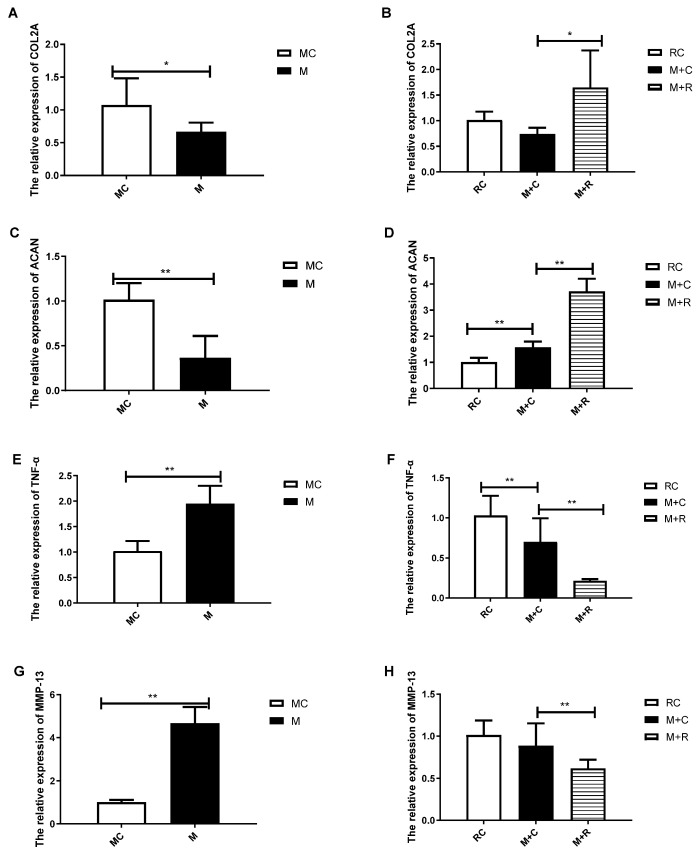
The gene expression of metabolic and inflammatory factors of knee joint cartilage. (**A**,**B**) the expression of COL2A mRNA; (**C**,**D**) the expression of ACAN mRNA; (**E**,**F**) the expression of TNF-α mRNA; (**G**,**H**) the expression of MMP-13 mRNA (n = 8, * *p* < 0.05, ** *p* < 0.01.)

RT-qPCR was used to detect the mRNA levels of COL2A, ACAN, MMP-13, and TNF-α in the knee joint cartilage of all groups. As shown in Figure 4, the gene expression levels of COL2A and ACAN in the M group were significantly lower than those in the MC group, while the expression of MMP-13 and TNF-α were significantly higher than in the MC group. The above results indicated that after 4 weeks of HITE, the cartilage catabolism in the M group was enhanced, and the cartilage was in a state of diffuse inflammation.

After HITE, the mice in the M + R group were subjected to 4 weeks of MITE, while a non-intervention strategy was employed for the mice in the M + C group. The results showed that the expression of ACAN in the M + C group was significantly higher than in the RC group. Compared with the M + C group, the expression of COL2A and ACAN in the M + R group increased significantly, while the expression levels of proinflammatory factors TNF-α and MMP-13 decreased significantly. This revealed that the MITE was better than the non-exercise option in terms of promoting cartilage synthesis, inhibiting cartilage decomposition, and reducing inflammation.

### 3.5. The Effects of Exercise on the Expression of miR-214 and Related Downstream Genes in Articular Cartilage

**Figure 5:**
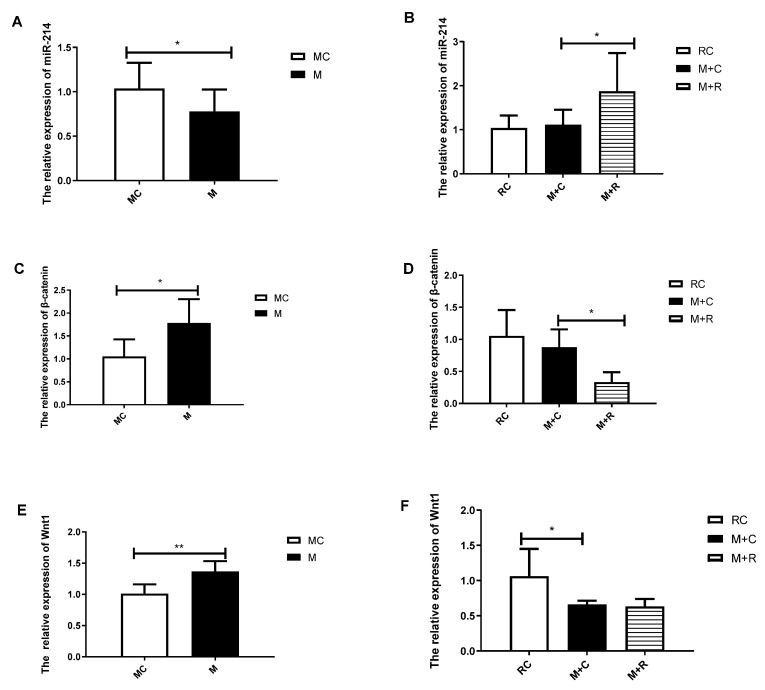
The expression of mouse cartilage miR-214 and its related downstream genes. (**A**,**B**) the expression of miR-214; (**C**,**D**) the expression of Wnt1 mRNA; (**E**, **F**) the expression of β-catenin mRNA. (n = 8, * *p* < 0.05, ** *p* < 0.01.)

After the total RNA had been extracted, reverse transcription was performed using an miRNA reverse transcription kit, and then RT-qPCR was used to detect the expression of miR-214. The results (Figure 5) revealed that the expression of miR-214 in the M group was significantly decreased, while the expressions of Wnt1 and β-catenin were upregulated in the MC group. Compared with the M + C group, after 4 weeks of MITE, the expression of miR-214 in the M + R group significantly increased, while β-catenin showed a downward expression. In summary, during the HITE period, the expression level of miR-214 in knee joint cartilage keep decreasing, while the expression of Wnt1 and β-catenin kept increasing. However, MITE was shown to partially reverse the expression of these genes.

### 3.6. Toluidine Blue O Staining and Chondrocyte Identification

**Figure 6:**
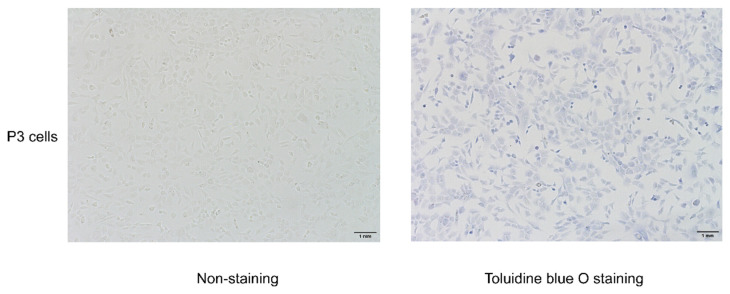
Toluidine blue O staining for chondrocytes.

Primary cells were passaged to the third generation for later intervention. P3 cells were evaluated with toluidine blue O staining to ascertain that they were chondrocytes. As shown in Figure 6, the acid-aggregated proteoglycan of chondrocytes can be stained by combining with the cations in toluidine blue O, which can stain the nuclei blue. The cells were polygonal, paving-stone shaped, which is the typical morphology of chondrocytes; thus, the cells were confirmed as chondrocytes.

### 3.7. The Cell Viability of Chondrocytes Stimulated with IL-1β at Different Concentrations

**Figure 7:**
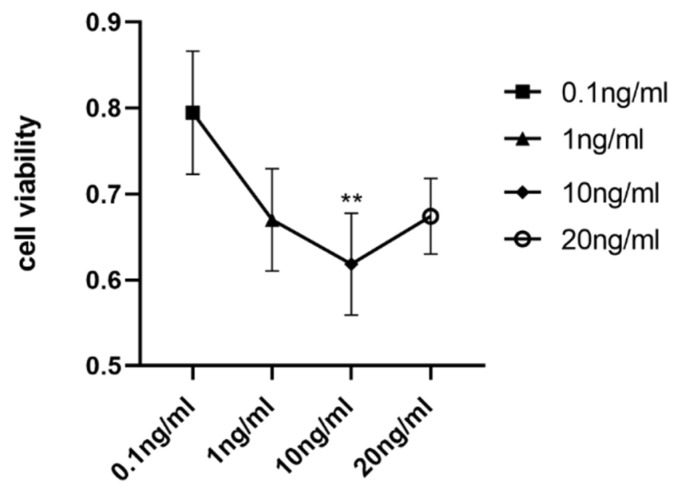
Cell viability of chondrocyte stimulated with IL-1β at different concentrations: comparison between 0.1 ng/mL and 10 ng/mL. (** *p* < 0.01).

Chondrocytes were stimulated with IL-1β at different concentrations (0, 0.1 ng/mL, 1 ng/mL, 10 ng/mL, 20 ng/mL) to induce inflammation and apoptosis. The CCK8 assay was used to determine cell viability and the best concentration of IL-1β to suppress cell viability. As indicated in Figure 7, the viability decreased most at a concentration of 10 ng/mL. Thus, a 10 ng/mL concentration of IL-1β was used for further experiments to induce inflammation and apoptosis in chondrocytes.

### 3.8. The Effects of Mechanical Strain on the Expression of miR-214 and OA-Related Genes in IL-1β-Treated Chondrocytes

**Figure 8:**
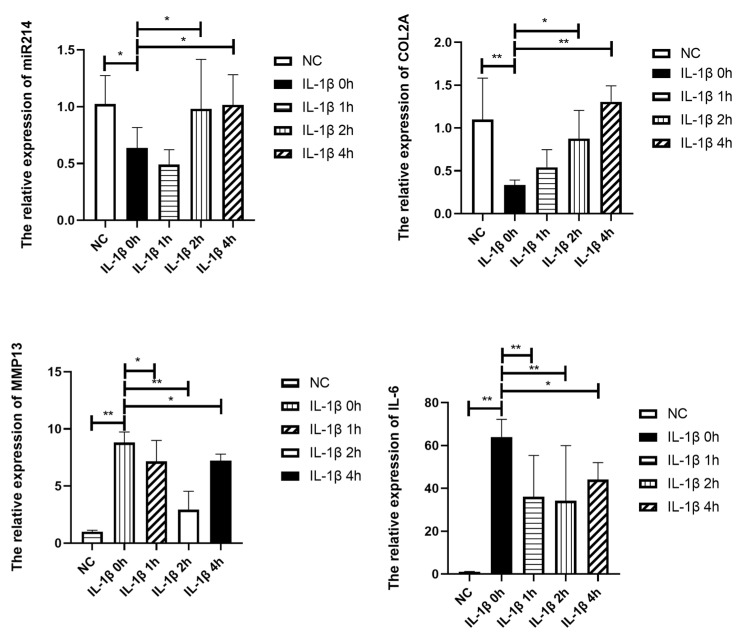
RNA expression of metabolic, inflammatory factors and miR-214 of chondrocyte. NC: Control group, IL-1β 0 h: IL-1β+ 0 h strain, IL-1β 1 h: IL-1β+ 1 h strain, IL-1β 1 h: IL-1β+ 1 h strain, IL-1β 2 h: IL-1β+ 2 h strain, IL-1β 4 h: IL-1β+ 4 h strain (n = 4, * *p* < 0.05, ** *p* < 0.01.)

**Figure 9:**
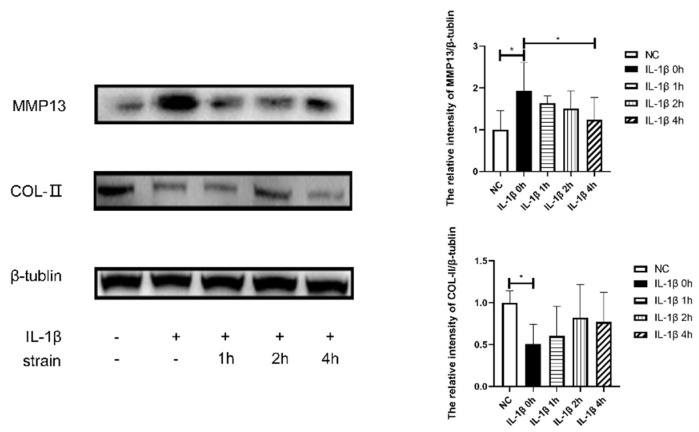
Protein expression of metabolic factors of chondrocytes. NC: Control group, IL-1β 0 h: IL-1β+ 0 h strain, IL-1β 1 h: IL-1β+ 1 h strain, IL-1β 1 h: IL-1β+ 1 h strain, IL-1β 2 h: IL-1β+ 2 h strain, IL-1β 4 h: IL-1β+ 4 h strain (n = 4, * *p* < 0.05).

As depicted in Figure 8 and Figure 9, 10 ng/mL of IL-1β induced a significant reduction in the effect of COL2 on mRNA and protein levels, which indicated reduced anabolism of chondrocytes. At the same time, the protease MMP13 and proinflammatory factor IL-6 were highly expressed. In conclusion, the stimulatory effect of 10 ng/mL of IL-1β on chondrocytes can lead to reduced anabolism, vigorous decomposition, and diffused inflammation, consistent with OA. The OA-like cells also showed a dramatic reduction in miR-214 expression (*p* < 0.05).

The application of 10% elongation mechanical strain reduced the catabolic and inflammatory state of IL-1β stimulated chondrocytes as the expression of MMP13 and IL-6 dramatically declined. There was a significant increase in the effect of COL2 on RNA (*p* < 0.05) and protein levels. Mechanical strain significantly increased the RNA expression of miR-214. In conclusion, 10% strain was shown to relieve the OA-like symptoms and increase the expression of miR-214, similar to the effect of MITE on PTOA mice.

## 4. Discussion

Under normal physiological load, the articular cartilage in the knee joint endures pressure, shearing force, and other complex mechanical stimuli while maintaining the dynamic stability of catabolism and anabolism within the cartilage matrix. However, excessive mechanical load on the articular cartilage reduces the anti-load and anti-distortion biomechanical capabilities of cartilage, making it unable to withstand the mechanical stress of normal load, resulting in cartilage damage and deformation. HITE can act on the cartilage matrix by exerting excessive mechanical load and causing the degeneration of articular cartilage [10,30,31]. We found that after 4 weeks of HITE, the cartilage volume decreased and calcification occurred in the M group. The histological evaluation results showed that the Mankin and OARSI scores of the M group were significantly higher than those of the MC group; the higher the score, the more severe the cartilage damage. In addition, RT-qPCR results showed that the expression of OA-related inflammation and catabolism factors in the M group were significantly upregulated, while the expression of anabolic indicators was significantly downregulated. Our results proved that 4 weeks of HITE could induce articular cartilage damage in mice; this was confirmed by histomorphological evaluation and gene expression analysis.

The subchondral bone is located under the articular cartilage; it contains osteoblasts, osteoclasts, and osteocytes. It can support the overlying articular cartilage. In normal conditions, the subchondral bone cushions the mechanical stress stimulation of the joint, thereby protecting the overlying articular cartilage. Articular cartilage also plays a role in buffering mechanical stress and reducing the mechanical load on the subchondral bone. As a functional unit, the two parts work together to maintain the homeostasis of joints. According to Wolff’s law, the trabecular bones are arranged in the direction of stress; they become thicker as the tension on them increases. The HITE imposed excessive mechanical load on the joints, which initially led to cortical bone thickening and increased bone mass. Our study found that the changes in subchondral bone in the M group were consistent with the early changes in bone morphology under high-load stimulation. The thickness of bone trabecula and cortical bone increased significantly in the M group. In addition, the BMD, BV/TV, Tb.N, and Tb.Th values of subchondral bone in the M group were significantly higher than those in the MC group, while the Tb.Sp value was inferior to that in the MC group. The Tb.Sp value represents trabecular bone separation; a high value indicates increased bone resorption and occurrence of osteoporosis. BMD, BV/TV, Tb.N, and Tb.Th are positively correlated with bone mass. Therefore, after 4 weeks of HITE, the bone mass of the M group increased significantly, which was consistent with the early adaptation of the subchondral bone under the high-load stress stimulation. An increase in bone mass in the subchondral bone can enhance the biomechanical properties of bone tissue, thereby enhancing the ability to cushion the mechanical load experienced by the overlying articular cartilage. The chondroprotective effect may be a compensatory response to articular cartilage damage under HITE.

MITE acts on cartilage by applying suitable stress stimulation to increase the synthesis of the cartilage matrix and inhibit the expression of catabolic and inflammatory factors [10]. This leads to gradual correction of the abnormally differentiated phenotype of chondrocytes and to the improved viability of chondrocytes, thus alleviating the progression of OA. Studies have found that MITE improves the microstructure (collagen fiber angle, PG content, and permeability) of the cartilage matrix and effectively restores the mechanical capabilities of articular cartilage [23]. Therefore, MITE can act on chondrocytes and the extracellular matrix of articular cartilage by applying appropriate mechanical loads to promote cartilage repair. In this experiment, 4 weeks of MITE was used to perform rehabilitation training on PTOA modeling mice. The results showed that, compared to the M + C and RC groups, the mice in the M + R group had less cartilage damage and calcification, and both Mankin and OARSI scores were significantly lower in the M + R group. The cartilage RT-qPCR detection results showed that the mRNA expression of OA cartilage damage-related genes such as TNF-α and MMP-13 was significantly lower than that of the M + C group, and the mRNA expression of cartilage synthesis factors such as ACAN and COL2A was significantly higher than that of the M + C group. These results proved that MITE could alleviate cartilage damage at the levels of tissue morphology and gene expression. To better determine a suitable mechanical load for rehabilitating OA, we designed an in vitro experiment. To this end, we added 10 ng/mL of IL-1β to induce inflammation and apoptosis in chondrocytes in order to mimic the state of OA chondrocytes. Then, 10% elongation mechanical strain last for 0, 1, 2, and 4 h was applied to the IL-1β stimulated cells. The effect of 10% mechanical strain on IL-1β treated cells was consistent with that of MITE on PTOA mice. The result showed that the application of 10% mechanical strain relieved the highly expressed IL-6 and MMP13 and increased the key component of the cartilage matrix (COL2). This certified that a suitable mechanical load (MITE or 10% strain) could have a rehabilitative effect on OA.

Many studies have found that articular cartilage degeneration, osteophyte hyperplasia, and abnormal bone remodeling of subchondral bone in OA mice are significantly inhibited when mice are subjected to MITE [23,34]. In our study, the Micro-CT results showed that the bone trabeculae in the M + R group were arranged neatly, and the thickness of the bone trabecula was significantly greater than those of the M + C and RC groups. The results of the bone morphology index showed that the Tb.N and BV/TV of the M + R group were also significantly higher than those of the M + C group, while the Tb.Sp was significantly lower than that of the M + C group. OA is known to be a chronic degenerative joint disease. In the early stages of the disease, there are abnormal changes in the functional activity of osteocytes, osteoblasts, and osteoclasts, resulting in subchondral bone resorption and osteoporotic bone density reduction [35,36]. In our experiment, the subchondral bone of the M + C group had an abnormal bone mass decline, which was detected by micro-CT evaluation. This was consistent with the early symptoms of OA. Compared with the M + C group, the bone mass of the subchondral bone in the M + R group after 4 weeks of MITE was significantly reversed. This result proved that 4 weeks of MITE could act on subchondral bone and normalize the abnormal bone remodeling. Wolff’s law states that “the trabecular bones are arranged in the direction of stress, and the trabecular bones become thicker as the tension on them increases”. The conclusion of Wolff’s law, i.e., increasing through use and decreasing through neglect, could explain the increase of bone mass in the M + R group. Zhou et al. reviewed the crosstalk between cartilage and subchondral bone and confirmed that articular cartilage and the subchondral bone are an integral functional unit [37]. The destruction of the buffer performance of subchondral bone will aggravate the aberrant stress and damage cartilage. In addition, the abnormal remodeling of subchondral bone leads to a bidirectional exchange of biochemical molecules in the bone-cartilage unit and promotes the development of OA. This experiment proved that 4 weeks of MITE can effectively block the abnormal bone remodeling of the subchondral bone and increase the bone mass. The increased bone mass further strengthens the support of the subchondral bone relative to the articular cartilage, which reduces the impact of external load on cartilage and plays a role in its protection. In summary, this experiment proved that 4 weeks of MITE not only relieves the abnormal inflammatory catabolism of articular cartilage, but also corrects abnormal subchondral bone remodeling.

Studies have found that the overexpression of miR-214 leads to chondrohypertrophy and tidemark advancement, resulting in a decrease in cartilage volume and degeneration. It was confirmed that miR-214 inhibited the development of OA in mice and exerted chondroprotective effects through the MAPK signaling pathway [38]. The specific regulatory role of miR-214 in articular cartilage has not been elucidated. Our study found that after 4 weeks of HITE, the expression of miR-214 in the M group was significantly lower than that in the MC group, while after 4 weeks of MITE, the expression of miR-214 in the M + R group was increased significantly, in contrast with that in the M and RC groups. After 4 weeks of rest, the expression of miR-214 in the M + C group was partially restored—in contrast to that in the M group—but without statistical significance, compared with the RC group. In addition, the expression of miR-214 in the M + C group was also significantly lower than that in the M + R group with MITE. It is speculated that the changes in the expression of miR-214 in the M + C group may have been due to the spontaneous recovery response of the body after the decrease in miR-214 expression during the 4 weeks of HITE. Based on this spontaneous recovery, the high expression of miR-214 in the M + R group was mainly attributed to the promotion of miR-214 expression by MITE. In vitro experiments showed that the expression of miR-214 was also significantly decreased in IL-1β 0h group compared with the control group. With the application of mechanical strain on the IL-1β stimulated chondrocytes, miR-214 expression was restored, similar to the effect of MITE on PTOA mice in terms of restoring the expression of miR-214. Thus, miR-214 may exert a chondroprotective effect and alleviate the progression of OA.

One study found that during the process of osteogenesis, miR-214 inhibited the differentiation of mesenchymal stem cells into osteoblasts by directly targeting the 3′UTR of β-catenin [39]. The Wnt/β-catenin pathway mainly activated β-catenin to transfer it into the nucleus to perform biological functions. Inhibiting the expression of β-catenin can inhibit the standard Wnt/β-catenin signaling pathway. Buchtova et al. found that the Wnt/β-catenin signaling pathway may be involved in the regulation of chondrocyte differentiation [40]. The excessive activation of the Wnt/βcatenin pathway induces OA-like changes in chondrocytes, which are mainly manifested by increased expression of COLX and MMP-13 and increased chondrocyte apoptosis [41]. In addition, the overexpression of Wnt inhibits the synthesis of ACAN in cartilage and contributes to OA [42]. Our experiment found that the expression of Wnt1 and β-catenin in mice increased significantly after HITE, and the expression of β-catenin was partially reversed after MITE. Therefore, we speculated that miR-214 exerts a chondroprotective effect by targeting β-catenin. The two processes, i.e., cartilage damage induced by HITE and partially reversed cartilage damage by MITE, may be closely related to the miR-214/β-catenin axis. On the other hand, studies have found that miR-214 can mediate bone formation and resorption, affecting the bone metabolism balance [43,44]. A corrupted metabolism balance of subchondral bone would lead to the development of OA [37]. MiR-214 may play an important role in both subchondral bone and articular cartilage, thereby influencing disease development. Unfortunately, this experiment failed to determine the relative expression of miR-214 in subchondral bone or to verify the direct correspondence of the miR-214/Wnt/βcatenin axis. Further experiments are needed to explore the specific mechanism of the miR-214/β-catenin signal and the expression of miR-214 in the subchondral bone to better describe the effect of miR-214 on OA.

## 5. Conclusions

In HITE-induced PTOA mice, the expression of miR-214 in articular cartilage was shown to decrease. MITE could partially reverse the cartilage damage induced by excessive exercise and increase the expression of miR-214 in articular cartilage. The results obtained from an in vitro experiment also confirmed that the miR-214 expression was decreased in IL-1β stimulated chondrocytes, and that the application of mechanical strain can restore the levels of miR-214. In conclusion, a suitable mechanical load can increase the expression of miR-214 and exert a chondroprotective effect against the development of OA.

## Figures and Tables

**Table 1:** Experiment grouping.

Groups	Abbreviations	Intervention Program	Sacrifice Age
Model control group (n = 8)	MC	4 w nonintervention	25 w
Model group(n = 8)	M	4 w HITE	25 w
Rehabilitation control group (n = 8)	RC	8 w nonintervention	30 w
Model + rehabilitation group (n = 8)	M + R	4 w HITE + 4 w MITE	30 w
Model + convalescent group (n = 8)	M + C	4 w HITE + 4 w nonintervention	30 w

**Table 2:** Treadmill exercise protocol.

Experiment Stages	Speed	Frequency	Slope	Time of Duration
Pre-adaptation	0–20 m/min	60 min each time; 5 times a week	5°	1 w
HITE Acceleration phase	0–20 m/min	10 min each time; 5 times a week	5°	4 w
HITE Uniform phase	20 m/min	60 min each time; 5 times a week	5°	
HITE Deceleration stage	20–0 m/min	10 min each time; 5 times a week	5°	
MITE	8 m/min	40 min each time; 5 times a week	0°	4 w

**Table 3:** Primer sequence.

Primer	Forward Sequence (3′→5′)	Reverse Sequence (3′→5′)
miR-214	ACAGCAGGCACAGACAGGC	
U6	AACGCTTCACGAATTTGCGT	CAGAAGGAGGAGGCAGGAAGAGG
β-catenin	CCCAGTCCTTCACGCAAGA	CCCTCTGAGCCCTAGTCA
COL2A	TCGGCCCTCATCTCTACATC	GGCTCCCAGAACATCACCTA
MMP-13	ACGTGTGGAGGTGAGGCATCC	GCAGAAGGCAGACCGCAATGG
TNF-α	AGGCTGCCCCGACTACGT	GACTTTCTCCTGGTATGAGATAGCAAA
IL-6	CTGCAAGAGACTTCCATCCAG	AGTGGTATAGACAGGTCTGTTGG
ACAN	CACTCCTGCCTGCTATGGAATGC	CCTGGTGATGCTGGTGCTGTTAG
WNT1	ACAGCGTTCATCCTCGCAATCACC	AAATCGTTGTTGTCACTGCAGCCC
β-actin	CGTTGACATCCGTAAAGACC	AACAGTCCGCCTAGAAGCAC
GAPDH	GTGTGAACGGATTTGGCCG	CCAGTAGACTCCACGACATA

**Table 4:** Mechanical straining protocol.

Group	Abbreviation	Strain Intensity	Time	IL-1β Stimulation
Control group	C	-	-	-
IL-1β + 0 h strain group	IL-1β 0 h	-	-	10%IL-1β
IL-1β + 1 h strain group	IL-1β 1 h	10%	1 h	10%IL-1β
IL-1β + 2 h strain group	IL-1β 2 h	10%	2 h	10%IL-1β
IL-1β + 4 h strain group	IL-1β 4 h	10%	4 h	10%IL-1β

**Table 5:** Morphological changes of subchondral bone in the femoral condyle of knee joint.

Group	BMD/mg HA·ccm^−1^	BV/TV/%	Tb.N/mm^−1^	Tb.Th/mm	Tb.Sp/mm
MC	1007.017 ± 5.65	0.599 ± 0.02	6.48 ± 0.188	0. 110 ± 0.002	0.137 ± 0.006
M	1024.669 ± 13.75	0.636 ± 0.02 *	6.58 ± 0.18	0.124 ± 0. 011	0.133 ± 0.008
RC	1034.46 ± 5.78	0.62 ± 0.02	6.72 ± 0.22	0. 115 ± 0.006	0.130 ± 0.006
M + C	1042.02 ± 7.86 ^#^	0.59 ± 0.02 ^##^	6.42 ± 0.42 ^##^	0. 111 ± 0.005	0.137 ± 0.009 ^#^
M + R	1056.40 ± 6.75	0.64 ± 0.02	7.14 ± 0.19	0. 121 ± 0. 009	0.128 ± 0.006

n = 8, * *p* < 0.05; comparison between the M + R and the M + C groups: ^#^ *p* < 0.05, ^##^ *p* < 0.01.

## Data Availability

Not applicable.
